# Mr. Headly, on Ophthalmia

**Published:** 1810-08

**Authors:** Henry Headly

**Affiliations:** Cambridge


### *To the Editors of the Medical and Physical Journal.*

Gentlemen,

As Ophthalmia, commonly called Egyptian Ophthalmia, has been so prevalent in the army these last six or seven years, and has of late made its appearance in private life, I conceive it a duty to communicate any thing that appears to lead to a more successful treatment of it. The generally received opinion is, that it was communicated by contagion from those troops who suffered so generally in Egypt, and since their return. A disease so novel and destructive, has naturally created much anxiety and investigation into its sources and mode of treatment; and the profession are indebted to Dr. Vetch for the able and accurate history he has given of the disease, as it appeared in the 52d and other regiments.

That it is contagious, Dr. V. considers certain, from the time of its appearance and other causes he assigns; but he observes, " the situation of Hythe barracks, the hot season of the year at which the disease made its appearance, from the exercise which the men daily underwent, exposed to the reflexion of the sun's rays, from the shingle and the great quantity of fine sand, which a long prevalence of blowing weather carried from it, a ready explanation of the origin of the disease seemed to present itself; yet as other regiments similarly situated and exposed, were not affected, these causes did not appear satisfactory. In another passage, he also mentions the disease prevailing chiefly in the neighbourhood of the Delta; and that moisture does contribute to the aggravation of it, as most strikingly observed in the increased and diminished severity of the symptoms, on the removal of the regiment from Riding-Street barracks to Maidstone, and its return to the former quarters, which are situated in a most extensive Marsh.

I do not mean to deny its contagious character, but I wish to draw the attention of others to the above causes, that it may be farther ascertained whether they can give rise to the disease, or excite it into action, or aggravate it in rendering the symptoms more violent, or in changing the character of the disease from the simple continued inflammation of the eye, to the more formidable intermittent form it has of late assumed. For in no one instance that has come under my observation, was I able to trace any contagious source.

The

The first time the disease came under my treatment was last summer, when Surgeon to the Cambridge Regiment of Militia; the greater number of cases were slight, and gave way to the usual simple treatment; other cases more active required general blood-letting, &c.; no doubt was entertained but it arose from the prevalent windy weather blowing up much fine sand and dust, accompanied with heat and reflexion of the sun's rays. After thirty or forty men had been attacked, the regiment then stationed at Yarmouth, removed to Chelmsford; the weather continued unusually hot and dry, and the men were daily attacked; after two or three weeks, the weather became cool, and much rain fell, (June) and about this time, two or three that had been attacked, and were considered convalescent, relapsed, and assumed the intermitting form with all its formidable train of symptoms. Bleeding largely, with purgatives, &c, and the antiphlogistic regimen, were employed without arresting the progress of the disease; in one case ulceration took place, and the other ruptured without ulcerating. I also had an opportunity of seeing several unfortunate cases at the Ophthalmia Depôt, Danbury barracks.

A short time after this, (in July) I was in town, and called on the late eminent Mr. Saunders, and related the symptoms and mode of treatment pursued; he said he had seen the 52d when labouring under the disease, and regretted circumstances prevented him staying with them so long as he wished; a great many cases had, however, since come under his treatment, and he was of opinion, bleeding, though sometimes necessary, was in many subjects attacked with the disease inadmissible; and mentioned that he had given bark in some strong marked cases with immediate relief from the paroxysm, &c.

On my return to the regiment, the two men in whom rupture and ulceration had taken place, continuing subject to the paroxysm. I immediately ordered pulv. cort. per ʒi. 2da. q. q. hor. sum.; and with the exception of one slight paroxysm, they had no farther return. I have since had several cases in private practice, which I will merely give an outline of; Dr. V. having so fully described the symptoms and treatment, it will be unnecessary to detail them.

T. M. Esq. was attacked on the 17th of May with slight inflammation of the right eye, and had two irregular paroxysms of pain, after which it became better, and the left eye was attacked; he applied a weak solution of ceruss. acetat.

20th and 21st. He had paroxysms in the evening.

22d. I was requested to put some leeches on at 4 P. M. the eye was then free from pain, but extremely vascular, and discharged tears copiously, with some purulency, and considerable sense of heat; the leeches afforded no relief, and the paroxysm occurred with increased violence at six o'clock; about eight I saw him, and ordered a draught with tinct. opii, gt. 40; this rather mitigated the pain, which continued six hours, when it subsided, leaving a great sense of heat, and constant discharge of tears, scalding the cheeks as they passed over.

23d. Ten o'clock A. M. has had occasionally a throbbing pain since six, and now feels sense of heat and enlargement, &c. as yesterday. Capt. haust. aper. cont. lot. saturn. Requested to be sent for next paroxysm occurring.

24th. Four o'clock A. M. paroxysm had been on an hour and a half. V. S. ℥xxx.; syncope took place, and he was relieved from all symptoms as long as it continued, but on recovering, sense of heat returned. Appl. emp. canthar. inter. scapul. Three o'clock P. M. another paroxysm has occurred, and been on him two hours. Capt. tinct. opii, gt. 40. A short time after, the pain subsided; but early in the morning (one o'clock) of the 25th, it occurred more severe, and lasted six hours, leaving an intolerable sense of heat, tears, and matter flowing copiously, from which, local applications afforded no relief. I now resolved to purchase a clear intermission by another bleeding, and throw in the bark. I accordingly opened a vein, and after taking about twenty-four ounces of blood, I wished to have desisted, if the pulse had fallen, or he felt the least faint; but these did not take place till near forty ounces were drawn; he then became faint, and soon afterwards extremely sick and low; coffee and a little wine were given, after which he fell asleep, and in five hours from drawing the blood, the sense of heat slightly returned. I immediately commenced giving decoct. cort. ℥ij. c. extr. cort. ʒß. every hour, and continued it for twelve hours, when it was given every two hours.

In the evening of the 26th, a little febrile heat was observed; and as it increased, the bark was desisted from, and a draught with aq. amm. acet. c. spt. æth. nitr. substituted during the night, and in the morning he recommenced the decoct. c. extr. every two hours.

28th. Free from pain and sense of heat; discharge of tears and pus less. Cont. decoct. cort. c. extr. 4ta. q. q. hor. After this time the eyes became gradually less vascular, cular, discharge of tears and matter decreased, and the patient gradually, though slowly, recovered perfect vision.

Case II.—Mr. T. Jun. Opened the bowels, applied leeches, and used saturnine lotion without relief; the paroxysm lasted four hours; on its recurrence, gave a draught with tinct. opii æther a. gt. xxx; this relieved the pain, after which I commenced giving pulv. cort. p. ℈ij. 2da. q. q. hor. and he had no farther return.

Case III.—Mrs. M's paroxysms had occurred three times, lasting two hours; the bark was here given as in the former case, and she had only one slight attack after.

Case IV.—Mary Papworth had been five weeks subject to slight paroxysms, lasting two hours, and complete opacity of the left eye had taken place. The bark was here given with immediate relief, absorption took place, and she has perfectly recovered the sight.

In the above cases, there was no constitutional affection discoverable by any symptom, the disease was confined entirely to the eyes.

The pain, as described by the patients, darts, throbs, and shoots with a sense of weight and fulness.

In the first case, I regretted bleeding a second time very much, but the symptoms were so formidable and urgent, I was fearful of a rupture taking place; and I did not think myself justified in giving the bark till a clear intermission took place. Bleeding, from the observation I have made, will arrest the symptoms, but does not prevent the recurrence; indeed, I am disposed to think the succeeding ones have been more violent, in consequence of the large depletions, and I shall be induced in any future case (where opium will not succeed) to try the effects of large nauseating doses of antimony in gaining an intermission, afterwards giving the bark.

I might have enumerated more fully the symptoms and the appearances of the eyes under the disease, but as I could have made no improvement or addition to the accounts published, I did not think it necessary. I only wish to draw the attention of medical men, more particularly in the army, to the administration of bark in the intermittent form of ophthalmia, in the full confidence of its success.

I am, &c.

HENRY HEADLY.

*Cambridge, July* 14, 1810.

